# Review on microgrids design and monitoring approaches for sustainable green energy networks

**DOI:** 10.1038/s41598-023-48985-7

**Published:** 2023-12-08

**Authors:** Ijaz Ahmed, Muhammad Rehan, Abdul Basit, Hasnain Ahmad, Waqas Ahmed, Nasim Ullah, Marian Piecha, Vojtech Blazek, Lukas Prokop

**Affiliations:** 1 Department of Electrical Engineering, Pakistan Institute of Engineering and Applied Sciences (PIEAS), Islamabad, Pakistan; 2 Department of Electrical Engineering, College of Engineering, Taif University, P.O. Box 11099, Taif, 21944 Saudi Arabia; 3 Ministry of Industry and Trade, Prague, 11015 Czech Republic; 4 ENET Centre, VSB-Technical University of Ostrava, 708 00 Ostrava, Czech Republic

**Keywords:** Energy science and technology, Electrical and electronic engineering, Energy infrastructure

## Abstract

Microgrids are power distribution systems that can operate either in a grid-connected configuration or in an islanded manner, depending on the availability of decentralized power resources, such as sustainable or non-sustainable power sources, battery backup systems, and power demands. The extensive adoption of inverter-based systems poses numerous technological challenges, necessitating a centralized management system to assure the system reliability and monitoring of the energy delivery networks. Thus, this research begins by highlighting these significant obstacles and then analyzes the present-day advances in multilevel control architecture for delivering on promised functionality. This article also discusses the development of innovative control technologies, such as introducing collaborative distributed approaches and reducing conventional three-stage patriarchal administration to fewer stages of system integration and functioning.

## Introduction

Microgrids (MGs) deliver dependable and cost-effective energy to specified locations, such as residences, communities, and industrial zones. Advance software and control systems allow them to function as a single unit and to manage the demand and supply of energy in real-time^1^. Most notably, MGs benefit companies and clients by strengthening dependability by integrating mobility at the transmission system’s local layer, improving energy efficiency using dynamic demand management, and lowering greenhouse gas emissions^2^. MGs can integrate sustainable and conventional inverter-based systems (IBS) for dependable electrical energy delivery to regional clients. These components create a self-contained autonomous system that can operate independently or in parallel with the primary power grid, depending on the needs and goals^3,4^. Sustainable energy-based distributed MGs provide an opportunity to increase energy efficiency, improve energy security, and reduce environmental impact while providing economic benefits to local communities. Relying on the conventional power generation has several vulnerabilities that can impact energy security, financial stability, and public health and safety. Transitioning to renewable energy-based distributed MGs can help to address these vulnerabilities by providing energy independence, resilience, and environmental benefits. Additionally, decentralized power resources (DPRs) can provide numerous benefits, including energy security, economic savings, enhanced energy access, and ecological protection^5,6^. Nonetheless, renewable energy sources such as solar, wind, and hydropower are referred to as ”intermittent” because they rely on time-varying natural resources. This is a difficulty for the energy operators that preserve the resilience of the electrical network by balancing the electrical supply and demand in real-time. In addition, advancements in smart grid technology make it simpler for grid managers to foresee and regulate fluctuations in the amount of renewable energy produced. This improves the grid’s overall dependability and efficiency^7^. While MGs offer a promising option for incorporating sustainable DPRs, significant initiatives are needed to create sophisticated control mechanisms to guarantee MGs’ safe, reliable, and cost-effective functioning.

Several issues need to be addressed to realize the full potential of DPRs in MGs. Some of the most significant challenges are (1) power electronic connectivity regulation for infrequent as well as non-dispatchable sustainable DPRs^8^, (2) preserving energy equilibrium to ensure stable voltage as well as frequency levels on the grid^9^, (3) reducing disturbances caused by the occurrence of asymmetrical and non-convex demands in compact MGs^10^, (4) developing safe communication channels for MGs as a preventative measure against hackers^11,12^ and (5) realistic modeling imbalanced events and perturbations in MGs^13,14^. Current research has shown a significant progression towards decentralizing, distributed, and multilevel control systems that divide these operational functions among several units^15–17^. When more distributed generation (DG) units are added to MGs, each sub-grid can reach its own safety and regulatory goals with little help from other sub-grids. On the other hand, decentralized control schemes rely on synchronized communication between components to assign control functions. This keeps subsystems from becoming overloaded^18^. Once this synchronization is performed, centralized control architecture (auxiliary controls) can be applied.

Generally, DG units are linked to the MG system via power electronics-based devices. Depending on their role in the network, these devices must be controlled differently. Ensuring reliability and establishing precise power exchange between these units require a central control to regulate the potential difference and ampacity at the outputs of these devices (inverters)^19^. The earlier control approaches implemented for MGs mainly restricted themselves to traditional droop approaches for power electronic coupling DPRs^20^. Such schemes were based on the premise that the production susceptibility of a converter is entirely inductive. Traditional droop was shown to be insufficient for accomplishing correct dynamic energy sharing when system impedance discrepancies existed among converters that were linked in a parallel configuration. As a result, various scholarly studies have suggested alternative versions of standard droop approaches to accomplish this control goal^21–23^. MGs based on geographically scattered inverters require complex control strategies to address their coherence and synchronization limitations. Hence, it leads to an increase in the adaptation of new non-droop data-driven control strategies^24–27^.

Intermediate control in an MG refers to the procedures that regulate the MG’s power flow to maintain stability and dependability. Secondary control occurs at the hardware level, keeping the overall production and demand harmony. Secondary control’s primary objective is to preserve steady frequency and voltage levels inside the MG by controlling the power production of various DPRs, including solar panels, wind turbines, and energy storage systems. The secondary control system continuously checks the power consumption and supply in the MG and regulates the production of the DPRs to guarantee that the total power produced is equal to the power consumption^28^. Distributed secondary control techniques with data transmission have replaced the centralized MG controller (CMGC) used in conventional secondary control^29^. Tertiary control, applied for maximizing the system’s profitability worldwide, might also be included in CMGC. It interacts only with upstream infrastructure to facilitate workable MG by allocating resources most effectively^30^.

The purpose of this research is to present an overview of the development of control methods in MG and to conduct a systematic evaluation of the various strategies for MG control that have been proposed in the published literature. Many publications have covered the numerous MG control techniques in depth^31–34^. Several system-of-systems (SoS)-oriented discussions on MG control techniques are found in^35^; nevertheless, the primary emphasis is on adapting SoS to MGs. The researchers of^5^ covered the fundamentals of MGs while also addressing emerging issues and potential from various fields, including law, economics, and regulation. MG control innovation was reviewed in^36^, with an emphasis on energy storage facilities (ESFs). A comprehensive literature analysis on the fully functioning-based taxonomy of MG control was presented in^37^. In light of the growing interest in MG control, this study presents a concise assessment of the state of the art in MG controls, as well as a discussion of its implications and the obstacles standing in the way of further study. The scope of this study includes the following:

1. Intermittent renewables problems, issues with the reliability of power and frequency, potential difference irregularities, and cyber-security problems are only some of the constraints and implications that sustainable energy MG has on the larger energy grid.
2. It describes advanced centralized control techniques like multi-agent systems oriented control (MASOC), model-predictive control systems (MPCSs) at the converter and strategic levels, consensus-oriented approaches, and machine learning-enabled monitoring approaches.
3. It identifies and classifies different control approaches from current research into one of the following control stages: (1) primary control, (2) secondary or intermediate control, and (3) tertiary or auxiliary control. Moreover, this categorization is helpful for scholars because it allows them to distinguish between control values, based on the time length at which their operations occur and the architecture criteria they must meet.
4. It assesses the difficulties experienced in MG control to suggest potential research topics.

The remainder of the study is structured as follows: “The theory of MGs” covers the fundamentals and designs of MGs. The third section discusses the issues associated with sustainable MGs integrated within the energy-delivering facilities, while “Prerequisites for DPR implementation in grid codes” summarizes IEEE DG connectivity criteria. “Advance hierarchical control” describes multilevel MG control, incorporating all conventional connectivity standards and resilient control levels to minimize connectivity problems. The last section summarizes the research.

## The theory of MGs

MG is a decentralized energy network that can function independently or in cooperation with a broader electricity network^38^. MGs are DPRs that include photovoltaic power, wind generators, batteries for storing energy, and backup systems, and are linked to a smaller-scale distribution network. MG systems offer greater autonomy over energy production and transmission to provide dependable, economical, and sustainable energy to the local population. Independent of the primary power grid, MGs can function in “island mode”, running their own systems and providing their own ancillary services like peak shaving and demand management^39^. Production resources, ESFs for smoothing out power oscillations and unbalance, demands, and a point of common connectivity (PCC) for joining and detaching MGs from the primary network to let it function in grid linked manner or islanded operation are all parts of a typical MGs design^40^. In addition, an MG central controller (MGCC) is often set up to regulate the MG power equilibrium and DPRs effectively. Micro sources track DPR functioning states and send that data to the MGCC, which then uses that data to send out minimal set-points to the individual DPR controllers^41^.

**Figure 1:**
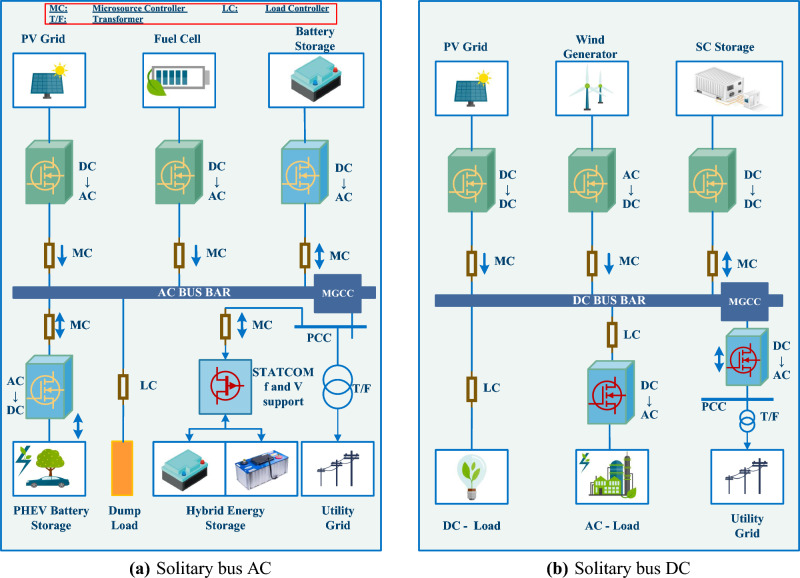
MG designs with a solitary bus.

**Figure 2:**
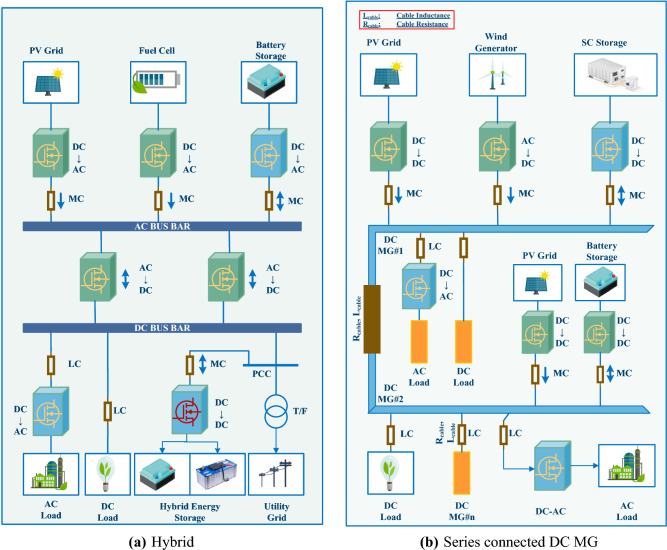
MG designs with multi-bus.

The dimensions, power supply, control technique, and function of MGs are just a few of the many ways they can be categorized. MGs can be either alternating current (AC), direct current (DC), or a mix of the two, depending on their resources, how much power they use, and how it gets to them. The literature, however, provides many detailed and extensive MG architectural models. For example, the models of MGs designs can be seen in^42^, wherein three feeds with delicate demands and the ability to island from the network through a fixed flip were addressed. It has a number of non-critical demands that can be met even if something goes wrong, as well as four micro-sources that can be controlled by peer-to-peer techniques. In^43^, the investigators provide an identical design wherein sustainable DPRs supply essential and non-critical home demands via three distribution system. MGs with a solitary bus architecture^34^, such as those depicted in Fig. 1a,b, are widely deployed because of their suitability for small and intermediate voltage systems. Besides (a) multi-level architecture for covering bigger regions with spatially dispersed DPRs^44,45^ and (b) multiple-bus design for increasing dependability in an urban region^46^, there are additional possible generalized MG architecture. Both of them are depicted in Fig. 2a,b.

Since AC MGs are already compatible with the architecture of existing AC networks, most development has been focused on learning more about and growing these systems. A more accurate distribution of reactive and active power, as well as enhancements in electric quality and frequency control, is among these advancements. In terms of dependability and efficiency, DC MGs are superior to AC MGs. Because DC MGs lack the concerns associated with imbalances, synchronization, and harmonics, they are not affected by these problems. Among the developments made in DC MGs are the implementation of synchronized control schemes, dependable power administration, and a set of voltage-regulating algorithms. Hybrid MGs have both alternating and direct current transmission systems, and DPRs possess direct current and are connected to an identical power system. As energy-storing devices and the DPRs are easily linked to the hybrid power grids network, there is less need to coordinate. Therefore, hybrid MGs are an attractive approach to merging future alternative power DPRs and e-mobility with minimal alteration to the existing transmission system and to lower total expenses^47^.

Even though MGs are distinguishable from the rest of the transmission network due to their stability, their control and operation are complex. Depending on their architecture and function, they can function in various ways. The most prominent modes are black start mode, grid support, grid connection, and island mode^16^. The primary constraints and objectives for micro-assets, demand controllers, and MGCCs are to transfer surplus energy or acquire inadequate energy via the converter in a grid-connected manner and to manage frequencies and voltages in stand-alone mode to achieve the regional balance of power.

## Problems and implications of MGs powered by sustainable sources on the power network

### Uncertainty of sustainable energy sources

The uncertainty of the technologies brought about by IBS in MGs has resulted in major difficulties in the functioning of the electrical system, despite the fact that these advancements have made enormous strides. There is a possibility that the amounts of energy production would change, which would make the MG vulnerable to frequency as well as voltage fluctuations at PCC. Additionally, as such incorporation of the unpredictability of wind power in MGs increases, the adverse effects of power surges in the middle frequency band (0.01 and 1 Hertz) become more pronounced^48,49^. Therefore, control mechanisms that have an essential function in the operation of MGs by guaranteeing continuity and dependability need to be developed to mitigate the effects of these connectivity complexities^34,50,51^. The uncertainty handling strategies can be broken down into eight distinct classes, which are as follows:

1. Incorporating a variety of various forms of sustainable power, all of which will supplement the others to achieve an improved power profile^52,53^.
2. Allowing power transmission in both directions across MGs for energy planning^54^.
3. Using energy storage devices to flatten out the production of sustainable power sources and significantly reduce intermediate frequency variations to ranges of 19–38%^55–57^.
4. Designing dynamic control techniques for decoupling linked to wind farms to satisfy power system criteria^58^.
5. Allowing for demand reduction to optimize power equilibrium^59^.
6. Integration of dispatchable units to offer support during times of low production from sustainable energy resources^60,61^.
7. Applying MPC-based normalized wind energy supply to power networks to minimize reduced frequency fluctuations related to intermediate band wind energy, which has a higher oscillation rate^62^.
8. Using hybrid approaches integrating two or more techniques to mitigate unpredictable power^63^.

### Resilience of frequency and system inertia

A change in frequency has a direct relation (proportional) to the system inertia and also results from any discrepancy between power generation and consumption. These shifts in dynamic behavior are described by the following models (1)–(4).

1 $$\begin{aligned} I = \int {{R^2}dm = {R^2}M}, \end{aligned}$$

2 $$\begin{aligned} K.E = 0.5\left( {I\alpha _M^2} \right) , \end{aligned}$$

3 $$\begin{aligned} \frac{d}{{dt}} = 0.5\left( {I\alpha _M^2} \right) = {P_M} - {P_E}, \end{aligned}$$

4 $$\begin{aligned} J = \frac{{K.E}}{\omega } = \frac{{0.5\left( {I\alpha _M^2} \right) }}{\omega }. \end{aligned}$$

Synchronous machine inertia as well as kinetic energy are determined via mathematical expressions (1) and (2), wherein *R* is the radius of the synchronous units rotating portion, *M* is the units mass in kilograms, and $\alpha _M$ is the synchronous units relatively stable angular rotor velocity. The swing model in expression (3) indicates that the mean accelerating energy directly impacts the energy supply’s reliability. The $\alpha _M$ value shifts whenever there is a mismatch involving the mechanical $P_M$ and electric powers $P_E$. *J*, the inertia factor, is defined as the resilience towards frequency variations caused by conserved kinetic energy in rolling element bearings during energy discrepancies by model (4), and $\omega$ is the production capability of the energy network under consideration.In conventional energy networks, the kinetic energy of synchronous machines offers steadiness subject to grid disturbances or demand and production discrepancies, thereby restricting the change rate of frequency (CRF). Nevertheless, increased adoption of solar and wind IBS reduces the mechanical inertia, leading to an increase in CRF. The CRF defined by the following model (5) denotes frequency divergence (following a quick imbalance among production and demand) as

5 $$\begin{aligned} CRF = \frac{{\Delta P \times f}}{{2J \times \omega }}, \end{aligned}$$

where $\Delta P$ represents the disparities among production and demand and *f* represents the system’s frequency.

Frequency changes are more significant and could cause the demands or IBS to break when an electric grid contains a synchronous machine and a sustainable energy supply that does not add to the system’s stability. Thus, an MG with a high percentage of sustainable energy sources needs to operate reliably during a significant CRF. The authors of the study^64^ presented a thorough analysis of the many methods through which wind farms and specific wind turbines can implement frequency management control schemes. The *V*/*f* modal control suggested in^65^ has been used to maintain safe voltage and frequency levels in an MG. Because sustainable DPRs are non-linear, a battery is used to smooth out the resulting frequency fluctuations. Other methods to reduce MG lag time include

1. Control strategies for synchronous machines that utilized virtual inertia were adopted in integrators^66^.
2. Use of batteries for energy storage to aid in the frequency management of MGs powered by photovoltaic cells^67^.
3. An inverter’s energy phase can be regulated by simulating non-linear harmonics with the system frequency as that of the natural frequency^68^.

### MG system voltage stability

Long-distance power cables are the main cause of voltage instability in traditional energy grids. MGs have negligible voltage dips due to their short feeder linkages. Yet, there may be an increase in voltage instability challenges if MGs become more prominent in the transmission network of the present day. MGs nowadays experience voltage imbalances due to issues like extremely low stable and fluctuating voltages, poor synchronization of DPR power flow against voltage (QV) droop curves, the failure of IBS DPRs to keep a constant voltage throughout the battery bank, and the DPRs’ inability to accommodate for future load growth^69^.

Conventional energy grids manage reactive production by adjusting end voltages at the generation or adjusted demand. However, MGs respond to any system-wide alteration in the DPR node voltages. As network voltage adjustments are linked to decentralized energy resource (DER) voltage regulations, a well-coordinated set of DER QV curves is required to reduce voltage disparity between buses, limit reactive current flow, and damp voltage swings^70^. Certain aspects of an MG network degrade the aggregate voltage pattern and net power generation when interruptions occur^71^.

Conventional approaches to managing reactive energy exchange fluctuations across various DPRs in an MG use QV droop features. The following model in (6) can be used to express this^45^:

6 $$\begin{aligned} Q = \frac{\upsilon }{{{R^2} + {X^2}}}\left[ { - R{\upsilon _2}\sin \theta + X\left( {{\upsilon _1} - {\upsilon _2}\cos \theta } \right) } \right] . \end{aligned}$$

In Eq. (6), $\upsilon _1$ and $\upsilon _2$ represent the potential difference magnitude of two vertices in MGs infrastructures isolated by energized line impedance $Z=R+jX$, and $\theta$ represents the phase angle across the $\upsilon _1$ and $\upsilon _2$.

MGs with tiny levels of and substantial inductive injectors or feeders $X \gg R$ allow *R* to be ignored in favor of $\sin \theta \approx \theta$ and $\cos \theta \approx 1$^45^. Therefore, reactive energy is proportional to the potential, but the assumption that inductive links drive MGs is debatable because inverters might have different yield impedances. As a result, this standard droop process frequently fails to attain the anticipated precision level in distributing reactive energy. Several enhanced droop and non-droop monitoring strategies have been implemented for parallel-linked inverters in DG networks. Additional information on this topic will be provided in “Advance hierarchical control”. In addition, because voltage assessments change across the MGs network, they could not indeed be utilized to ensure a balanced distribution of global reactive energy. If the highest voltage dip of an MG network can indeed be specified by knowing the system parameters of that system, then the voltage pattern can be kept under reasonable parameters.

### Quality of power and oscillations

The prevalence of asymmetrical and fluctuating demands has increased the incidence of power quality issues in small-scale island-mode MG networks. As the harmonics produced by IBS grow to unacceptable levels, they lead to power outages, tripped circuit breakers, lost communications, and overloading. Supraharmonics (potential difference and current pattern disruption in the spectrum of 1–140 kilo-Hertz) are created when distributed sustainable energy supplies are present in an intermediate voltage system^72^. Harmonics output has been subjected to the rules and regulations established by the grid system to guarantee that the grid’s current and voltage patterns are compatible. Harmonic disturbance of voltage levels and current must be at most five percent according to all regulations and criteria, except for the more stringent rules in the UK, which mandate that present harmonics must be at most three percent^73^.

There are currently minimal restrictions or criteria for supra-harmonics in the energy grid. Because of this, additional research and studies into this topic are required to satisfy customers’ demands in a dependable and adaptable way. The researcher may find it helpful to consult publications^74,75^ for a comprehensive summary of the published literature concerning power quality reduction approaches.

### Problems with cyber security

A cyber system controls all networked data, networking systems, and physical power elements, including IBS, energy storage devices, and demands. The significant interdependence between those schemes increase the likelihood of problems, including transmission breakdowns, compromised data security, and excessive data management. Threats to MGs, analyzed through a cyber-physical network lens, have been the subject of multiple publications^76,77^. Researchers have addressed them as follows in^78^:

1. Cyberattacks through side channels allow an adversary to deliberately obstruct elements of a system’s adaptive processing or conceal power network disturbances.
2. Attacks, known as distributed denial of service (DoS), can cause authorized users to lose login to a network for an extended period.
3. Attacks against measures designed to leak private information, which, when successful, manipulate detecting or controlling data and cause the functioning of the physical process to become unreliable.
4. Threats posed by viruses and code, including Stuxnet, are increasing, causing portions of the MGs to self-destruct.
5. Attacks that involve the exploitation of information lead to weaknesses in the embedded devices of grids.

It is widely acknowledged that ensuring the safety of MGs, which can be considered for hybrid cyber-physical infrastructures, is a significant challenge. As a result, most recent research has concentrated on developing methods for identifying and isolating a malicious activity. For example, the researchers in^79,80^ propose new collaborative algorithms for locating and minimizing the effects of cyber attacks, often known as “stealth attacks”, in the auxiliary frequency as well as voltage regulation sublayers, respectively. Model (7) demonstrates that cooperative voltage, as well as frequency auxiliary regulators, can accomplish the following operational objectives under common functioning situations:

7 $$\begin{aligned} \mathop {\lim }\limits _{x \rightarrow \infty } {M_y}\left( t \right) = {M^*},\mathop {\lim }\limits _{y \rightarrow \infty } {W_{av}},d{c_i}\left( y \right) = W_{DC}^* \end{aligned}$$

In Eq. (7), $M^*$ is the required worldwide system frequency and $W_{DC}^*$, $W_{av}$, and *y* are the appropriate global network voltage, average reference voltage, and associated layer. This metric may identify cyber attacks like denial of service and blocking. However, stealth attacks can sneak into the network without alerting users and compromise various sensors and communication channels. Nevertheless, in the event of a cyber attack, the consensus mechanism built on cooperation shifts to model (8) as

8 $$\begin{aligned} M_y^f\left( t \right) = {M_y}\left( t \right) + yM_y^a,\mathop {\lim }\limits _{y \rightarrow \infty } {V_{av}},d{c_i}\left( y \right) = V_{DC}^a. \end{aligned}$$

In Eq. (8), $y=1$ shows attack component with the notation $M_y^a$ and $V_{DC}^a \ne V_{DC}^*$. However, it does not provide an adequate condition to determine the junctions of an attack, as every remnant matching requires a global knowledge. In this scenario, a sign of an attack is the controller’s effort at setting the final value to a fixed reference voltage.

As a result, mitigating the effects of stealth operations on frequency and voltage require both a collaborative security for vulnerabilities element and an event-driven robust control system^81,82^. A supplementary frequency monitoring built on a persistence measure is a method made by the researchers of^83^ for islanded AC MGs. According to the findings, there is a one-to-one relationship between improving the durability score and improving the susceptibility of convergence to cyber attacks that involve inserting false data. In addition, the denial of service, replay, and false data or information injection attacks have been modeled and analyzed for potential effects on the system characteristics of volts, frequency, and proactive and reactive energies in isolated AC MGs in^84^. The frequency network was shown to be the highest sensitive form of data link, while the reactive energy line was found to be the lowest susceptible. The MG network will remain stable thanks to a supplementary controller based on a flexible transmission line. This will continue till the cyber attack has indeed been located and eliminated. The first strategy for coping with cyber challenges in MGs includes finding and countering cyber-security attacks. The second strategy includes establishing decentralized adaptable control schemes for minimizing the negative impacts of malicious attacks without sensing, classifying, and then attempting to remove or restore the vulnerable agents^84,85^. Identifying and countering cyber-security attacks becomes a way to solve cyber problems in MGs.

Yet, MGs require a self-healing capability to continue functioning even when under attack. In order to restore a system in the modern era, the restoration process must be fully autonomous and programmed, unrelated to any transmission system operator (TSO)^86^. Plug-and-play management and transient reliability of converters are necessary for development, however, grid-forming configuration switching may be autonomous at the inverter level. Maintaining MG functioning requires grid-level cooperation and an understanding numerous power sources working in concert.

## Prerequisites for DPR implementation in grid codes

At the delivery level, classical electrical energy systems (EES) lacked backup and active production facilities. Yet, as more and more MGs are connected to the power grid, there will be an increased demand for power system professionals to ensure the continued efficiency and security of the system. The Institute of Electrical and Electronics Engineers (IEEE) is developing the necessities and processes necessary to properly incorporate DPRs into current energy systems to homogeneously supply consumers’ energy demands.

The methods^87,88^ aim to establish two essential functions; the delivery function, which is necessary for computing and providing predefined DPRs and variable load demand, and the conversion function, which is tasked with handling the switch among grid-engaged and standalone modes. The following are the most essential DPRs functional topics^89,90^:

1. Reactive power management and voltage regulation
2. Production of reactive power
3. Flexibility in voltage and frequency of the power system
4. Capacity to withstand faults and disturbances
5. Energy reliability in the power system.
6. Isolation and safety measures

**Figure 3:**
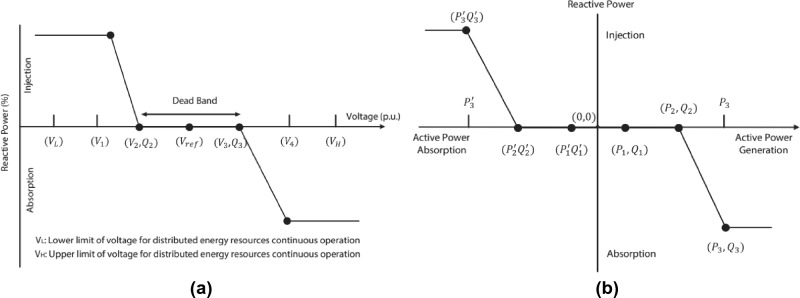
DPRs distinctive voltage-reactive-power profile^34,91^.

The DPR’s primary duty under the design codes is to maintain a constant voltage through the management of active and reactive allocation. By maintaining the power flow inside the potential limitations as stipulated by the TSO, the IBR facility must maintain the power factor consistently. The system ought to be capable of regulating the flow of reactive power in response to changes in voltage (VQ mode) as well as active power (PQ mode) (please refer to Fig. 3a,b)^34,91^.

DPRs are required to infuse (over-excited) or collect (under-excited) reactive energy inside the dynamic power potential limits defined in Table 1 for active output power ranges up to the highest limit^92^. The classes provide specific attributes and capacities for voltage fluctuations within the acceptable range of operation. These specifications apply to DPRs classed under standard performance requirements Class 1 and 2. The operational limits correspond to working at an active power output above five percent but below twenty percent of the rated active power. In this range, the DPRs must be able to exchange reactive power up to the lowest level specified in Table 1. This minimum value is determined by multiplying the active power output by the ratio of the minimum reactive power to twenty percent of the designed active power. The numbers 44 and 25 in the column indicate the ability to absorb reactive power, expressed as a percentage of the device’s nominal apparent power rating (kVA). This is the second network code criterion.

**Table 1:** The lowest potential for injecting and absorbing reactive power.

DPRs class	Range of operation for active power (Pr) in percentage of nominal value $(\%)$	Potential for injecting reactive power in percentage of nominal value $(\%)$	Potential to tolerate reactive power in percentage of nominal value $(\%)$
1	5 < Pr < 20	44	25
2	5 < Pr < 20	44	44

The least consistent active power potential (Pmin) or 5% of recommended active power (Prated) can always be exceeded by the delivered active power (kW). TSO can control the need for reactive electricity both regionally and globally. Reactive energy production during significant DPR integration falls within class 2, whereas the lowest productivity criteria for class 1 can be found in moderate DPR participation.

The acceptability criterion for both voltage and frequency on the system mandates that DERs must continue functioning even if the utility’s voltage and frequency fluctuate within a specific range of values. Nonetheless, IBS units are disconnected due to grid disruptions. IBS units must maintain their functionality as if they were active production units to prevent the sudden disengagement from disrupting the electric grid. The three possible voltage levels, (a) zero-voltage, (b) low-voltage, and (c) high-voltage, are included in the FRT requirement’s scope^92^. Functional parameters for a DPR beyond the ongoing operation zone are defined by the first, second, and third atypical functioning behavior classes, respectively. For example, the requirements for extensive connectivity of DERs are specified under Class First atypical operational behavior.

In the IEEE benchmark^92^, topics such as DC current infusion limits, voltage spikes contributions, abrupt voltage fluctuations, strobe generation at PCC, and periodic current disturbance are discussed. DPR should restrict DC current infusion to 0.5% of the maximum nominal generating current. The mean RMS voltage variation rate above one second at PCC’s intermediate voltage range must not exceed 3% the standard level per second. Step or gradient variations in RMS voltage shall remain less than 5% of the standard level per second during a one-second over time, and the DPR must enforce this restriction for reduced voltage performance. Distortion product ratios are restricted in how much they can distort currents and the overall allowed current. As DPR is an active screen, the electrical grid administrator and DPR administrator can collaborate on any additional settings.

The MG equipment’s standalone and safety needs necessitate the transfer operation’s effective performance, avoiding destroying or producing an electromagnetic arc to the MG components. The transfer operation supervises the functioning of the control scheme throughout transitioning between grid-connected, standalone, and reconnected modes. It controls migration for many situations: (1) islanding by design, (2) islanding by chance, (3) reintegration, and (4) black start. Under purposeful islanding, IEEE benchmark^92^ necessitates the DPR to de-energize and break within 2–5 s of settlement period. The many essential typical prerequisites for DPRs are failure ride-through and safety functionality.

## Advance hierarchical control

**Figure 4:**
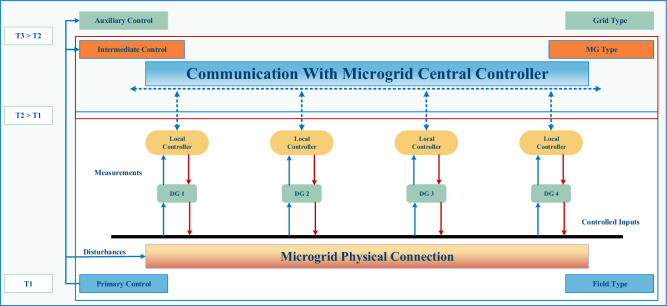
Hierarchical MG control design.

Because MGs come in many different shapes and sizes, their controllers must be strong, flexible, and capable of essential computations at high speeds. Using a hierarchical control scheme with multiple time levels is especially interesting when you think about how fast things are for managing outcomes and how slow things are for economic scheduling. An MG controller design with data transmission, a global controller, and localized control systems can be centralized or decentralized. An MG with centralized control uses a transmission network and a central hub. An MG with decentralized control uses local data and cooperation between micro-sources, capacity controllers, and MGCCs to control tasks. While a centralized server maintains global control performance by being aware of every network location, the vulnerability of massive-scale MGs to single-point breakdowns and under attacks renders the adoption of centralized processors impractical^93,94^. On the same point, an entirely distributed strategy cannot be implemented since special hardware is too expensive. As a result, a modern mix of the two strategies is adopted. This strategy is known as multilevel MG administration, consisting of three operational layers, as illustrated in Fig. 4: (1) primary (field type), (2) intermediate (MG type), and (3) auxiliary (grid type).

Hierarchical MG control systems have been chosen as the primary control method. Still, the changing behavior of alternative energy DPRs and demands makes it hard to set limits on hierarchical control. So, decentralized and distributed strategies are being used to make it easier to use MG control systems^95,96^.

Recent research suggests how the traditional three levels of the hierarchical management scheme are combined into smaller systems to realize plug-and-play functionality^97^. Optimal control designs, also known as laminar monitoring designs, were stated in^98^ for the development of distributed equipment and networks by incorporating limitations on data flow among layers (formation of control schemes and segmenting targets into sub-tasks). Such integrated control systems reduce control messages and adequately express control algorithms between systems while still optimizing. However, integrating levels to improve MGs’ speed, effectiveness, and acceptability requires further investigation. Even so, hierarchical MGs control is still the best^99^, as flexible control structures and precise control mechanisms are still being made. Hierarchical control attains a good balance between fully centralized and distributed control mechanisms and manages many customizable parts while meeting strict quality goals. It has a dependable and robust networking system and compatible routing protocols. Thus, the subsequent sections elaborate on these three control layers (primary, intermediate, and auxiliary).

### Primary-level control

Primary-level control systems consist of localized controllers. These controllers govern energy distribution across micro-sources, regulate the current and voltage characteristics of digital power inverters, and maintain the frequency of the entire system. It also serves as the initial line of defense against electrical voltage and frequency fluctuations in an MG, and it works on the slowest time scales^95^. By using neighborhood data, variations can be reduced to a minimum; therefore, a communication network may or may not be needed (see Table 2).

Current source converters (CSCs) and voltage source converters (VSCs) are two kinds of power semiconductor inverters that may be used to transform the alternating or direct current from clean energy sources^47^. A grid-forming VSI regulates potential differences and system frequency, making it well-suited for MGs operating in an isolated configuration. In contrast, a grid-following VSI regulates both reactive and active energy.

However, control of inverters is required for controlling production characteristics of both voltage and current, in addition to the proper proportions of outer feedback loops, i.e., droop or non-droop-based regulation and maximum power point tracking^100^. The subsequent section analyses in further depth the inner control dynamic characteristics of such inverter classes and the outside energy-sharing control approaches.

**Table 2:** Analysis of primary class (droop and non-droop) energy exchange approaches.

Approach	Refrances	Summary	Key characteristics
Conventional droop	^101^	Converter are linked together in parallel and regulated by production frequency as well as volt droop	Systems having cascading feedback control, connectivity autonomy and extremely adaptive
Angle droop	^102^	The amplitude and phase of the voltage regulate the active and passive components of energy	A greater level of droop yield could compromise system stability The technique can be modified to handle power imbalances
Virtual frequency droop	^103^	Produced by rotation of an orthogonal structure at a certain position	Resistive lower voltage MGs can effectively isolate voltage and frequency, but the control technique must account for DGs’ functional limits
Instant droop	^104^	By determining momentary virtual impedance using the droop indices, converters in DC MGs are able to share current	Algorithm successfully reduced cycling currents and ensured that all nodes received the same amount of power during intermittent events
Droop in several dimensions built on harmonics	^105^	The DC and AC components of a dual MG employ voltage level harmonic components	Reduced circulation currents, optimum energy exchange, and preserved voltage levels at standard
Virtual impedance droop	^106^	Virtual yield resistance, reduce power inefficiencies and distribute demand current across inverters	Reduced voltage MGs share energy proportionally
Decentralized adjustable droop	^107^	Voltage stability and demand balancing are accomplished by interconverter transmission	The computation ability to share demands equitably remains unaffected by interruptions in connectivity and the adjustment of virtual characteristic impedance
Steady droop regulator	^108^	For sustained energy exchange, the controller establishes DER baseline data	Control strategy was able to provide a seamless configuration shift and dampened transient response

### Grid-following: inner current control

**Figure 5:**
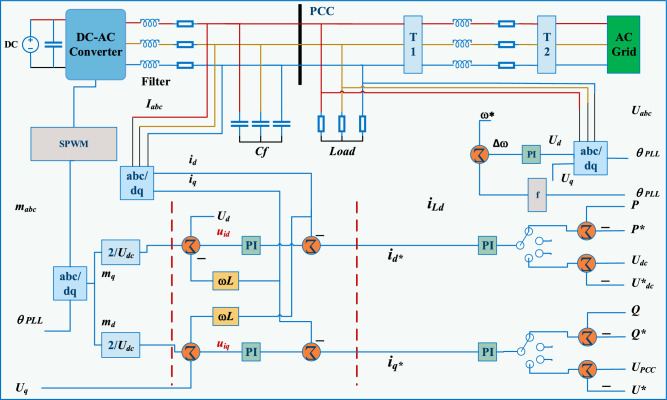
Grid-following control configuration.

A DPR integrated with a grid-following voltage source converter coupled to the power grid depicted in Fig. 5 demonstrates the corresponding 3-phase dynamical model represented by (9)^109^:

9 $$\begin{aligned} dt\left( {{U_{{c_{abc}}}} - {U_{PC{C_{abc}}}}} \right) = {L_f}d{i_{abc}} + {R_f}{i_{abc}}\left( {dt} \right) . \end{aligned}$$

For *dq* benchmark, the corresponding model for currents and potential difference is represented as given below:

10 $$\begin{aligned}{} & {} dt\left( {{U_{{c_d}}} - {U_{PC{C_d}}}} \right) = {L_f}d{i_d} + {R_f}{i_d}\left( {dt} \right) - co{L_f}{i_q}\left( {dt} \right) , \end{aligned}$$

11 $$\begin{aligned}{} & {} dt\left( {{U_{{c_q}}} - {U_{PC{C_q}}}} \right) = {L_f}d{i_q} + {R_f}{i_q}\left( {dt} \right) - co{L_f}{i_q}\left( {dt} \right) . \end{aligned}$$

Here (10) and (11) are the reference values for the *d*-axis and *q*-axis components of the output voltage respectively. The model (10) and (11) are linked with $co{L_f}$ and final potential is shown in model (12) and (13) as provided below:

12 $$\begin{aligned} {U_{cd}}= & {} {V_{id}} + {U_{PC{C_d}}} - co{L_f}{i_q}, \end{aligned}$$

13 $$\begin{aligned} {U_{cq}}= & {} {V_{iq}} + {U_{PC{C_q}}} - co{L_f}{i_d}. \end{aligned}$$

Here (12) and (13) are *dq*-frame voltages. The coefficients of the filter resistance and inductance are denoted by $R_f$ and $L_f$, respectively, while *co* indicates the system frequency.

### Grid-following: energy and potential control

For integrating energy from green sources (such as photovoltaic and wind power), the most common type of inverter used is a VSC that follows the grid or feeds electricity back into it. Its primary function is interacting with an alternating current system and trading reactive and active electricity. A VSC is often built to supply current. This paper also takes into account this design pattern. A power station or grid-forming inverter is necessary for island mode functioning of an MG because the MG cannot produce the required voltage as well as frequency without them^110^.

Reference currents $i_d^*$ and $i_q^*$ are often supplied by an energy regulator in grid-following inverters that regulate the energy sent to the system and consumed by the localized demand. The following expressions represent the power elements.

14 $$\begin{aligned} \begin{aligned} P&= {U_{PC{C_d}}}{i_d},\\ Q&= - {U_{PC{C_d}}}{i_q}. \end{aligned} \end{aligned}$$

In Eq. (14), *P* denotes active power, where *Q* represents the reactive power. As shown in Fig. 5, $i_q$ may also be employed to change the potential^111^.

### Grid-forming: inner potential control

**Figure 6:**
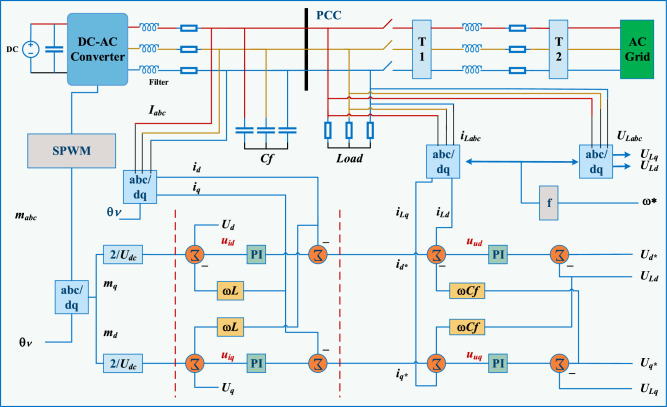
Grid-forming control configuration.

Figure 6 displays the control design of a grid-forming converter^109^, which is comparable to that of a grid-following converter except for the two circular cascaded cycles. An outside cycle monitors the benchmark voltages to compute current for an inside current control cycle. The dynamic model for grid-forming is given as below:

15 $$\begin{aligned}&{C_f}d\left( {{U_{Ld}}} \right) = dt\left[ {{i_d} - {i_{Ld}} + co{C_f}{U_{Lq}}} \right] ,\nonumber \\&{C_f}d\left( {{U_{Lq}}} \right) = dt\left[ {{i_q} - {i_{Lq}} + co{C_f}{U_{Ld}}} \right] . \end{aligned}$$

In model (15), $i_L$ and $v_L$ represents the demand current and voltage respectively.

### Grid-forming: outer droop control loop

**Figure 7:**
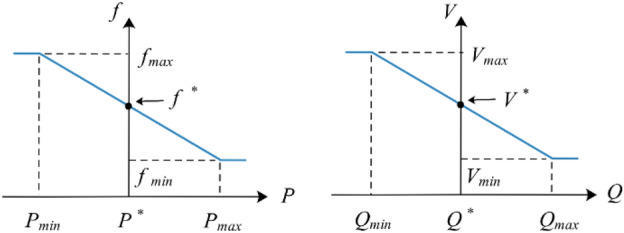
Typical features of a droop^109^.

The bulk of energy exchange strategies for grid-forming converters depend on droop management as an external feedback controller to maintain the standard MG frequency as well as voltage during PCC^112^ (see also Table 2). To accomplish that, we reduce the stable operation of synchronous generators by using linear exchange equations involving voltage (*V*), frequency (*f*), reactive power (*Q*), and real power (*P*). The visual correlation across the droop coefficients is shown in Fig. 7.

Energy distribution varies owing to demand and grid redesign^113^, however, it continues to be a complicated process to calculate the magnitude of this impedance. Thus, developing more complex droop mechanisms for precise reactive energy pooling is a current field of study. While these controls’ concepts, parameters, and used cases vary, a few examples are shown below.

1. A global droop mechanism in^114^ is considered so that neither impedance nor resistance can be overlooked any further. It is possible to eliminate the tight correlation across both energies by implementing a linear rotational mapping function to change active and reactive power.
2. A consensus-oriented decentralized voltage regulation is presented in^115^ for linked MGs that are managed by converters and include inductive energy cables and configurable power designs. The algorithm successfully regulated all variables’ amplitude and frequency to their rated set points, enabling it to perform precise reactive power distribution.
3. Harmonic energy exchange and eliminating swirling harmonic components are fixed using a consensus approach in^116^ that modifies the converter impedance and resistance outcomes.
4. A power factor orientation droop adjustment in^117^ is provided that is independent of power transmission impedance and demand kind when the system is operating in a standalone configuration. This control allows a seamless shift between the grid-connected model and the standalone design.

### Grid-forming: droop-less oriented control approaches

An increase in information exchange control techniques for inverter control^118,119^ can be attributed to address the shortcomings of droop regulation approaches, like sluggish dynamic response and laborious tweaking of control settings. Droop-less network-based control approaches for dynamic demands and unpredictable situations enhance frequency and voltage control. They depend on a data network to regulate the power produced by sustainable DPRs and may be deployed in a centralized or distributed design. One option is a bounded control setting approach that has reduced objective function computations and computation latency. Other applications involve advanced control approaches such as inductive reasoning, neural network computing, and evolutionary computing^120,121^.

On the primary layer of control, much research has been carried out, although they are still in the early stages. The primary purpose of this work is to update existing methods even though droop reduction solutions for MGs with many random components have already been the topic of substantial research. As a result, cutting-edge techniques such as the meta-heuristic algorithm described in^122^ are utilized to regulate the frequency and the voltage in an offshore MG in relation to the limits imposed by its operational capabilities.

### Control approaches at intermediate level

In the MG system, any power and frequency irregularities caused by primary control are brought back under secondary or intermediate control for correction. It functions on a shorter time scale to send task signals to the primary layer, which the primary element uses to manage the economy and synchronize MG networks with the utilities. Secondary administration can be implemented in either a hierarchical or distributed design. This helps to decrease distortions and energy imbalances by controlling DPRs in accordance with the primary regulators of said relevant devices (refer Table 3). In a configuration where the MG is controlled centrally, the MG administrator plays a pivotal role in synchronizing DPRs and achieving optimal MG performance^73^. A few examples are shown below:

1. Using a dominant micro station (master) to establish reference amplitude as well as frequencies for subordinate micro stations (slaves) is one example of the master-slave regulation method discussed in^123^.
2. Machine learning-based power administration approaches are described in^84,124,125^. These algorithms optimize MG operations, reduce grid electricity instabilities, extend the lifetime of battery storage, and harvest the highest amount of energy from sustainable DPRs.
3. An integrated voltage and frequency peripheral regulation is presented in^126^ to enhance the robustness and functionality of an isolated mode. A master MGs control system regulates the principal droop profiles of DPRs to restore equilibrium to the reactive and active energy and the voltage and frequency parameters.
4. An observer-based centrally located faulty damper secondary approach is presented in the reference^127^ for fault assessment and service denial.

Even though they work effectively in various functioning situations, these approaches are vulnerable to breakdown at a specific point since they rely heavily on centralized transmission. On the opposing side, the decentralized design divides the task of regulation optimizing between the allocation network provider, the MGs control system, and the local processors. Distributed secondary supervision approaches employ independent and specialized regional controllers who use neighbour data to lower the price of operations and the characteristics of the system that backs them up. For example, in^128^, a secondary control scheme that relies on two types of processors for every DG unit is presented. The mechanism can accurately share reactive energy, and return frequency as well as voltage to their reference values. Secondary control systems are positioned across the network connectivity, and the primary control system generates a command signal for the first layer. Further examples of this can be seen in Table 3.

**Table 3:** Analysis of secondary control approaches.

Approach	Ref	Outline	Aspects
Master–Slave govern and MG’s central controller	^129^	The MG central controller transmits commands to every unit according to supplied metrics like electricity, battery charge status, or DC linkage power	System of power administration that is innovative and durable, achieving constant efficiency and performing much under extreme varying load
Fuzzy logic controller	^130^	Preserve cell capacity and voltage equilibrium with flexible controller	Increasing the storing program’s longevity reduces variations in the amount of energy put into the electrical system, while also extending the storing device’s lifecycle
ANNs controller and RL	^131^	Sources involve MG component-specific data	Obtained optimum energy from DPRs and precisely managed energy with the least amount of acquired power
Centralized secondary administration	^132^	MG central controllers transmit predefined for turbines, the parameters of which are decided by a consensus procedure employing the Levenberg–Marquardt technique	The program functioned well in numerous infiltration and overload cases involving sustainable energy sources
Secondary regulation using composite agents	^133^	Control components of the system underneath a Hybrid MG centralized unit and secondary control enabled by multiple agents in islanded MGs	Connectivity breakdowns do not affect the energy quality or reliability of the system since the decentralized multi-agent system takes over for the centralized MG central controller
Multi-agent system	^134^	The goal of the MGs’ specialized agents is to reduce production expenses	In DPR MGs that relied on sustainable energy sources, voltage as well as frequency were accurately restored, energy usage tracked output, and bank demands dropped
Model predictive control	^135^	An innovative genetic algorithm-based control mechanism for MG networks, allowing for improvement of performance across a time scale of just 24 h	Improved calculation time and enhanced risk mitigation while dealing with MGs
Decentralized multi-agent systems	^136^	Data is exchanged across neighbouring devices in voltage regulation that use backstepping as well as movable MAS and NN techniques	Recovery of voltage was successful, and reliability was enhanced throughout a wide range of load circumstances, with the chattering problem being significantly decreased

### Control approaches at auxiliary level

As the uppermost level of governance in grid-connected MGs, tertiary or auxiliary control governs how the MGs communicate with each other and the upstream system. Table 4 lists the research on tertiary control techniques. For efficient scheduling, optimal performance planning, and controlling power flow in both directions between the power network and MG^30,137^, tertiary management functions are performed at the minimum time scale. Centralized tertiary regulation systems were more generally utilized to control MGs; however, in^138^, a highly decentralized tertiary controller has been established for every DG unit. Decentralized primary control systems (DPCS), decentralized secondary control systems (DSCS), and decentralized tertiary control systems (DTCS) are the three components that make up the unified supervision approach, which offers enhanced adaptability and dependability. A conventional droop mechanism is the foundation of DPCS, whereas a non-convex droop adjustment serves as the basis for DTCS. The control scheme is modelled by model (16), which has the following form:

16 $$\begin{aligned} {T_i} = {T_p}\left( {{P_i}} \right) + {T_s}\left( {\eta ,{T_{i,m}}} \right) + {T_t}\left( {\mu ,{P_i},{\psi _i}\left( {{P_i}} \right) } \right) . \end{aligned}$$

**Table 4:** Analysis of tertiary-level methods.

Approach	Ref	Outline	Aspects
Secondary authority based on master/slave and peer/peer relationships	^139^	Deliver optimal functioning of MGs by controlling various elements	Smooth transitions across grid-connected as well as standalone configurations, even in the connectivity breakdowns, are made possible by incorporating peer-to-peer control
Tertiary adjuster	^140^	Adjust the current and voltage MG factors to trade power needed at PCC	The synchronization of MG current and voltage corresponds with the intended energy sharing at PCC. Nonetheless, enormous imbalanced current passes if the circuit is poorly handled
Tertiary control using evolutionary algorithms	^141^	Global energy integrity is regulated by tertiary control depending on target mechanism and machine limitations	Imbalance system modelling and simplification enable economic improvement of energy allocation at an advanced rank
Decentralized control	^142^	The architecture relies on decentralized, every DG regional controllers	Phase regulation, optimal functioning of DGs, and enhanced MG behavior as well as reliability

The gain parameters for DTCS and DSCS are denoted by $\eta$ and $\mu$, while the yield frequency and observed frequency are indicated by $T_i$ and $T_{i,m}$. Several decentralized tertiary control systems are now working to handle a variety of MG difficulties, including black start functioning, reserve power planning, and general harmonic aberration adjustment^46,143^. Tertiary control also helps balance demand and makes switching between grid-connected and standalone operations easy. For example, the authors of^123^ describe a master-slave peer-to-peer fusion control method that allows smooth changes among the two operational modes of MGs. The MG controls frequencies and amplitudes under grid-connected functioning while keeping necessary energy production constant. MGs are more resilient because they use a peer-control strategy that takes precedence when the information network fails. Furthermore, in a three-phase delivery network, voltage fluctuations can be introduced by single-phase demands or asymmetrical electrical transmission. This could cause damage to voltage-sensitive devices. Tertiary supervision is a cost-effective method for balancing the restoration operations of numerous DGs. Further compensation devices, such as series or shunt-activated power diffusers in^144,145^, were introduced to minimize imbalance. The technique considers various lines and DGs’ compensating constraints and voltage stability requirements.

### Modern control approaches

According to existing scholarly publications, this part presents several contemporary control strategies. In contrast to traditional linear control systems, these approaches offer higher dynamic characteristics under all working situations despite disadvantages, including the stuttering problem and extensive mathematical formulation (for details, please see Table 5).

**Table 5:** Synopsis of principal features of control methods^146^.

Features	Linear	AI, ANNs and Fuzzy logic	Model predictive Control	Reinforcement learning	Consensus MASs
Control intricacy	Moderate	Strong	Limited-moderate	Limited online but stronger offline	Moderate to strong, based on connectivity structure and pattern of entity relationships
Design and variables	Not required	Required	Not required	Not required in prototype reinforcement learning	Not required
Limitations participation	No	No	Yes	Yes	Yes
Efficiency	Moderate	Significant	Superb	Sub-optimal	Superb

#### Introduction to multi-agent system (MAS)

To achieve the MG monitoring objectives, a multi-agent system (MAS) involving a group of autonomous agents can be employed^147,148^. Agents are distinguished in terms of MG security, control efficiency, and economic functioning by their independence, societal interactivity, responsiveness, and identity^149,150^. A decentralized MAS method may more easily share data and coordinate operations by dividing a vast power grid into smaller, more manageable components. A single controller governs similar units in a cooperative MAS system to draw inferences about their control systems. To sum up, MAS employs hierarchy to improve MG management in a dynamic context by classifying agents based on their respective power status. Entities in a MAS’s system primary, intermediate, and auxiliary levels undertake diverse control roles and rely on distinct information pathways to carry out their activities autonomously^151^. The accessibility of elements, the control functions performed by those agents, and the type of data provided between those agents affect the aggregate efficiency of the managed system. These three parts are controlled and built the same way as most MAS structures.

#### MPC-oriented approaches at grid level

MGs increasingly use MPC in their planning to overcome nonlinear economic optimization barriers^152^. Both the grid-level and converter-level variants of the MPC algorithm include a prediction framework, a solution mechanism, and an objective function. These three essential components are included in the grid-level version as well. Unlike its predecessor, this technique maximizes the MGs’ efficiency while considering several competing objectives and constraints^153^. For example, the work of^154^ presents an MPC-based regulator for home power systems with a distributed generation and storing system. The controllers perform storing device optimization, power consumption forecasting, and power trading.

Also, in^155^, researchers looked at how MPC can be applied to control backup devices in MGs. A solar energy system and rechargeable batteries in an ESS MG are managed using a combined MPC^156^. In^157^, an MPC-based technique is used to control a faster magnetic energy storage device so that changes in the unit’s electrical current and power don’t cause chromatic oscillations. When applied to voltage control with a DC/DC compressor in an SMES network in MGs, the proposed control approach can reduce eddy current losses for the DC power of a superconducting circuit, resulting in favorable effects.

#### AI-oriented control approaches

When problems with reliability and unpredictability are worst, more than logical analysis and computer simulations of MG networks are needed for reliable control systems. Intelligent controllers powered by artificial intelligence can adjust to complexities and do not necessitate any existing understanding of the system’s functioning. Thus, their potential use in MGs, including electricity supply and demand restoration, capacity proportioning, security, energy delivery optimization, and demand management, has garnered a lot of attention^158^.

The photovoltaic forecast program in^159^, an artificially intelligent neural network, allows MGs to be regulated in real-time. MG controllers use the predictions application’s outputs as one factor in making predictions about photovoltaic units’ electricity production. As a result, MG recovery mechanisms like fuel inverters only activate when required. In addition, the following are examples of other machine learning-based controllers used for MG electricity supply and consumption regulation:

1. A prospective controller powered by a cognitive system (ANNs) is used in^160^ to maximize the efficiency of simulated synchronous machines operating in low-voltage MG systems. The controller comprises an artificial neural network responsible for developing the conceptual framework and an optimizer to reduce the effect of anomaly.
2. In^161^, MG networks are controlled by an ANN architecture operating at the secondary layer. After experiencing perturbations such as network separation and variations in design variables (rotor gain, automated voltage converter yields, and cutoff frequency), the secondary regulator restores the steady state voltages and the system’s frequency.
3. A controller parameter set constructed using neural networks for MG groups to lower frequency variation is proposed in^162^. After being presented with approximately 5000 multiple combinations of arbitrary data for both input and output, the modified neural network improved the PID gain parameters for the lowest level of frequency fluctuation.

Integrated MGs have yet to be completely investigated despite the increasing significance of ANN-enabled solutions in the MG area. Hence, approaches for autonomous market pricing, energy pooling, and optimal energy supply control in networks of MGs must be investigated in upcoming studies.

#### Reinforcement learning (RL)-oriented approaches

Traditional controllers are no longer useful because of changes to the network, such as the redesign and integration of more dispersed generation and demand. MGs are particularly vulnerable to the effects of these occurrences since they happen so regularly. Improved agent behavior while optimizing the objective functions is the goal of sophisticated control mechanisms, including RL-oriented management approaches. Arithmetically, RL consists of a Markov chain of events with three independent variables: the actor, the incentive indication, and the surrounding context. An agent’s actions in the world have consequences, both deterministically and stochastically^163^. Some examples of RL-oriented approaches that have been used in the past are as follows:

1. Because they are exposed to distribution characteristic modifications, linked impedance, and capacitive demand fluctuations, the frequency indications of distributed generators can be quickly converged to the standard level of 50 Hz with an RL-oriented intermediate frequency regulation of MGs, as described in^164^. It is unnecessary to pump large amounts of energy into the network because the operational energy values are kept under the specifications.
2. For droop-controlled MAS-oriented mega MGs, the work in^165^ suggested a decentralized auxiliary control of frequency and voltage recovery depending on RL. Agents determine their regional voltage and frequency and share that data. The baseline settings of both frequencies and voltages are changed after 0.1 s, while RL-oriented methods keep the rest of the systems in check.
3. To research the delayed advantage-actor dissent-oriented MAS RL technique described in^166^, a four-line rechargeable ESD simulation was carried out. When harmonic frequency management and SOC balance synchronization can be done jointly in the face of DoS attacks, the suggested system can attain its full potential as a system.

#### Consensus-based leader and leaderless oriented approaches

The MAS idea was considered in MGs, and the consensus model has indeed been employed as the fundamental concept for cooperative control of bots^167^. Every agent uses the data available locally and the consensus algorithm to cooperatively collaborate with the neighbors in its neighborhood to arrive at a consensus^168,169^.

Second-level multilevel MG management has implemented a consensus technique to restore frequency and voltage characteristics in MGs. This is especially true when synchronizing grid building and grid tracking inverters, for which consensus-based collaborative control mechanisms have been developed. The authors of^138^ provide a joint control analysis of grid-forming and grid-following converters, which guarantees MG plug-and-play functionality and maximizes energy produced from sustainable sources. To bring the voltages of multiple DGs into agreement, we use a paradigm federalization technique to combine control conditions and design a global second-order feedback control technique.

Power administration in MG networks is another area where consensus supervision has been used. For instance, in^170^, the consensus concept has been used to address the energy dispatch issue for an MG with five lines using a quadratic objective model. To strike a good compromise between production and consumption, we determine the incremental cost that each device should update. Using a consensus mechanism to maximize the cost-effectiveness of MG electricity networks has much promise. How to use this concept to provide synchronization among many variable components, such as storage devices and generation machines, in an MG network is an intriguing area for further study.

## Conclusion

This study provided an overview of recent developments in microgrid administration and conducted an in-depth evaluation of the three layers of the hierarchical system: primary, intermediate, and tertiary. It established centralized, decentralized, and distributed control systems, comprehensively categorizing numerous control techniques reported in the research and placing them in these three groups. According to academic research, multiple droop-based and non-droop-based active and reactive energy pooling strategies exist at the primary level. However, there is still a potential for more development. In addition, secondary-level control was offered to detect secondary voltage and frequency faults, resulting in a network’s primary control and ensuring an efficient strategy. With either a centralized or distributed architecture, this was accomplished by the MGs’ centralized processors governing particular components of the MG network. Furthermore, tertiary-level used in a microgrid network was addressed, emphasizing its potential for establishing the MGs’ ideal power supply and achieving the most efficient system functioning. We accomplished this by synchronizing nearby MGs with upstream/distribution energy infrastructure while considering the characteristics of MG operations and other circumstances. It was also noted that using a mix of modern control methods is on the upswing, which bodes well for making MGs easier to regulate. Sustainable, resilient, and adaptable MGs developing and managing solutions are the way of tomorrow. Several factors, including the increasing prevalence of green energy sources, the development of modern storage methods for energy, and the rise of electric vehicles are shaping the power landscape. These shifts are being prompted by our shared duty to deal climate change and create a better tomorrow, as well as the demand for green and sustainable energy sources. As the vanguard of this revolutionary movement, MGs carry the potential of a more effective, environmentally sustainable, and robust energy ecosystem. Further investigation is required to ascertain whether developing cooperative control schemes and integrating hierarchical control levels may enhance functional resilience in advanced sustainable MG systems.

## Data Availability

The data-sets used and/or analysed during the current study is available from the corresponding author on reasonable request. All of data-set used in the study has been either provided or cited in the article.

## List of symbols

DPRs: Decentralized power resources
IBS: Inverter-based systems
MGs: Microgrids
CMGC: Centralized MG controller
SoS: System-of-systems
ESFs: Energy storage facilities
MASOC: Multi-agent systems oriented control
MPCSs: Model-predictive control systems
PCC: Point of common connectivity
DERs: Decentralized energy resources
TSO: Transmission system operator
AC: Alternating current
DC: Direct current
CRF: Change rate of frequency
DoS: Denial of service
EESs: Electrical energy systems
IEEE: Institute of Electrical and Electronics Engineers
RMS: Root mean square
CSCs: Current source converters
VSCs: Voltage source converters
DPCSs: Decentralized primary control systems
DSCSs: Decentralized secondary control systems
DTCSs: Decentralized tertiary control systems

## References

[1] Zheng X, Wu H, Ye Q (2022). A cloud fog intelligent approach based on modified algorithm in application of reinforced smart microgrid management. Sustain. Cities Soc..

[2] Daneshvar M, Mohammadi-Ivatloo B, Zare K (2023). An innovative transactive energy architecture for community microgrids in modern multi-carrier energy networks: A Chicago case study. Sci. Rep..

[3] Heidary J, Gheisarnejad M, Rastegar H, Khooban MH (2022). Survey on microgrids frequency regulation: Modeling and control systems. Electr. Power Syst. Res..

[4] Ahmed I, Rehan M, Basit A, Tufail M, Hong K-S (2023). A dynamic optimal scheduling strategy for multi-charging scenarios of plug-in-electric vehicles over a smart grid. IEEE Access.

[5] Norouzi F, Hoppe T, Elizondo LR, Bauer P (2022). A review of socio-technical barriers to smart microgrid development. Renew. Sustain. Energy Rev..

[6] Ahmed I, Rehan M, Basit A, Hong K-S (2022). Greenhouse gases emission reduction for electric power generation sector by efficient dispatching of thermal plants integrated with renewable systems. Sci. Rep..

[7] Tatar SM, Akulker H, Sildir H, Aydin E (2022). Optimal design and operation of integrated microgrids under intermittent renewable energy sources coupled with green hydrogen and demand scenarios. Int. J. Hydrogen Energy.

[8] Hu J, Shan Y, Cheng KW, Islam S (2022). Overview of power converter control in microgrids-challenges, advances, and future trends. IEEE Trans. Power Electron..

[9] Chopra S (2022). Power-flow-based energy management of hierarchically controlled islanded ac microgrids. Int. J. Electr. Power Energy Syst..

[10] Zhao Z (2022). Harmonics propagation and interaction evaluation in small-scale wind farms and hydroelectric generating systems. ISA Trans..

[11] Liu C, Wang X, Yao T, Wang X (2023). Self-triggered h infinite consensus-based secondary control of ac microgrids with uncertainty of communication. Int. J. Electr. Power Energy Syst..

[12] Basit A, Tufail M, Rehan M, Riaz M, Ahmed I (2023). Distributed state and unknown input estimation under denial-of-service attacks: A dynamic event-triggered approach. IEEE Trans. Circ. Syst. II Express Briefs.

[13] Farrokhabadi M (2019). Microgrid stability definitions, analysis, and examples. IEEE Trans. Power Syst..

[14] Ahmed, I., Rehan, M., Hong, K.-S. & Basit, A. A consensus-based approach for economic dispatch considering multiple fueling strategy of electricity production sector over a smart grid. In *2022 13th Asian Control Conference (ASCC)*, 1196–1201 (2022).

[15] Basit A, Tufail M, Rehan M (2022). Event-triggered distributed state estimation under unknown parameters and sensor saturations over wireless sensor networks. IEEE Trans. Circ. Syst. II Express Briefs.

[16] Modu B, Abdullah MP, Sanusi MA, Hamza MF (2023). Dc-based microgrid: Topologies, control schemes, and implementations. Alex. Eng. J..

[17] Basit A, Tufail M, Rehan M, Rashid HU (2022). A non-uniform event-triggered distributed filtering scheme for discrete-time nonlinear systems over wireless sensor networks. Trans. Inst. Meas. Control..

[18] Nudell TR (2022). Distributed control for polygeneration microgrids: A dynamic market mechanism approach. Control. Eng. Pract..

[19] Habibi SI (2023). Multiagent-based nonlinear generalized minimum variance control for islanded ac microgrids. IEEE Trans. Power Syst..

[20] Safamehr H, Izadi I, Ghaisari J (2024). Robust v–i droop control of grid-forming inverters in the presence of feeder impedance variations & nonlinear loads. IEEE Trans. Ind. Electron..

[21] Behera S, Dev Choudhury NB (2022). Sma-based optimal energy management study in a connected pv/mt/dg/v2g/bess/wt on ieee-33 bus considering network losses and voltage deviations. J. Inf. Optim. Sci..

[22] Ahmed I (2022). A novel hybrid soft computing optimization framework for dynamic economic dispatch problem of complex non-convex contiguous constrained machines. PLoS One.

[23] Mukherjee V (2022). Intelligent electric vehicles charging coupled demand response of isolated microgrid. Energy Stor..

[24] Lin, S.-W., Chu, C.-C. & Tung, C.-F. Distributed q-learning droop control for frequency synchronization and voltage restoration in isolated ac micro-grids. In *2022 IEEE Industry Applications Society Annual Meeting (IAS)*, 1–8 (IEEE, 2022).

[25] Ahmed, I. *et al.* Adaptive swarm intelligence-based optimization approach for smart grids power dispatch. In *2022 International Conference on Emerging Technologies in Electronics, Computing and Communication (ICETECC)*, 1–6 (IEEE, 2022).

[26] Khan MYA, Liu H, Shang J, Wang J (2023). Distributed hierarchal control strategy for multi-bus ac microgrid to achieve seamless synchronization. Electr. Power Syst. Res..

[27] Ahmed I, Rehan M, Basit A, Tufail M, Hong K-S (2023). Neuro-fuzzy and networks-based data driven model for multi-charging scenarios of plug-in-electric vehicles. IEEE Access.

[28] Sheykhi N, Salami A, Guerrero JM, Agundis-Tinajero GD, Faghihi T (2022). A comprehensive review on telecommunication challenges of microgrids secondary control. Int. J. Electr. Power Energy Syst..

[29] Aazami R, Esmaeilbeigi S, Valizadeh M, Javadi MS (2022). Novel intelligent multi-agents system for hybrid adaptive protection of micro-grid. Sustain. Energy Grids Netw..

[30] Babayomi O (2022). Advances and opportunities in the model predictive control of microgrids: Part ii-secondary and tertiary layers. Int. J. Electr. Power Energy Syst..

[31] Roslan M (2022). Microgrid control methods toward achieving sustainable energy management: A bibliometric analysis for future directions. J. Clean. Prod..

[32] Barik AK, Jaiswal S, Das DC (2022). Recent trends and development in hybrid microgrid: A review on energy resource planning and control. Int. J. Sustain. Energ..

[33] Ahmed, I. *et al.* Technological, financial and ecological analysis of photovoltaic power system using retscreen®: A case in Khuzdar, Pakistan. In *2022 International Conference on Emerging Technologies in Electronics, Computing and Communication (ICETECC)*, 1–6 (IEEE, 2022).

[34] Jain D, Saxena D (2023). Comprehensive review on control schemes and stability investigation of hybrid ac-dc microgrid. Electr. Power Syst. Res..

[35] Huang Y, Wang Y, Liu N (2022). Low-carbon economic dispatch and energy sharing method of multiple integrated energy systems from the perspective of system of systems. Energy.

[36] Lin X, Zamora R (2022). Controls of hybrid energy storage systems in microgrids: Critical review, case study and future trends. J. Energy Stor..

[37] Kanakadhurga D, Prabaharan N (2022). Demand side management in microgrid: A critical review of key issues and recent trends. Renew. Sustain. Energy Rev..

[38] Ahmed I, Basit A, Mustafa F, Alqahtani M, Khalid M (2023). The nexus of energy in microgrids: A review on communication barriers in distributed networks auxiliary controls. IET Gener. Transm. Distrib..

[39] Polleux L, Guerassimoff G, Marmorat J-P, Sandoval-Moreno J, Schuhler T (2022). An overview of the challenges of solar power integration in isolated industrial microgrids with reliability constraints. Renew. Sustain. Energy Rev..

[40] Tambunan HB (2023). Research trends on microgrid systems: A bibliometric network analysis. Int. J. Electr. Comput. Eng. (2088-8708).

[41] Zuo K, Wu L (2022). A review of decentralized and distributed control approaches for islanded microgrids: Novel designs, current trends, and emerging challenges. Electr. J..

[42] Fani B, Shahgholian G, Alhelou HH, Siano P (2022). Inverter-based islanded microgrid: A review on technologies and control. e-Prime-Adv. Electr. Eng. Electron. Energy.

[43] Kamal F, Chowdhury B (2022). Model predictive control and optimization of networked microgrids. Int. J. Electr. Power Energy Syst..

[44] Dragičević T, Lu X, Vasquez JC, Guerrero JM (2016). Dc microgrids-part ii: A review of power architectures, applications, and standardization issues. IEEE Trans. Power Electron..

[45] Fazal S, Haque ME, Arif MT, Gargoom A, Oo AMT (2023). Grid integration impacts and control strategies for renewable based microgrid. Sustain. Energy Technol. Assess..

[46] Yan L, Sheikholeslami M, Gong W, Shahidehpour M, Li Z (2022). Architecture, control, and implementation of networked microgrids for future distribution systems. J. Mod. Power Syst. Clean Energy.

[47] Shafiee-Rad M, Sadabadi MS, Shafiee Q, Jahed-Motlagh MR (2022). Modeling and robust structural control design for hybrid ac/dc microgrids with general topology. Int. J. Electr. Power Energy Syst..

[48] Fotopoulou M, Rakopoulos D, Stergiopoulos F, Voutetakis S (2022). A review on the driving forces, challenges, and applications of ac/dc hybrid smart microgrids. Smart Grids Technol. Appl..

[49] Ahmed I (2023). The role of environmental initiatives and green value co-creation as mediators: Promoting corporate entrepreneurship and green innovation. SN Bus. Econ..

[50] Mojumder MRH, Hasanuzzaman M, Cuce E (2022). Prospects and challenges of renewable energy-based microgrid system in Bangladesh: A comprehensive review. Clean Technol. Environ. Policy.

[51] Ahmed I (2022). Multi-area economic emission dispatch for large-scale multi-fueled power plants contemplating inter-connected grid tie-lines power flow limitations. Energy.

[52] Alizadeh A, Kamwa I, Moeini A, Mohseni-Bonab SM (2023). Energy management in microgrids using transactive energy control concept under high penetration of renewables; a survey and case study. Renew. Sustain. Energy Rev..

[53] Ren Y (2022). Optimal design of hydro-wind-pv multi-energy complementary systems considering smooth power output. Sustain. Energy Technol. Assess..

[54] Zhao J, Wang W, Guo C (2023). Hierarchical optimal configuration of multi-energy microgrids system considering energy management in electricity market environment. Int. J. Electr. Power Energy Syst..

[55] López A, Ramírez-Díaz A, Castilla-Rodríguez I, Gurriarán J, Mendez-Perez J (2023). Wind farm energy surplus storage solution with second-life vehicle batteries in isolated grids. Energy Policy.

[56] Ahmed I, Rao AR, Shah A, Alamzeb E, Khan JA (2014). Performance of various metaheuristic techniques for economic dispatch problem with valve point loading effects and multiple fueling options. Adv. Electr. Eng..

[57] Mehrasa M, Sheikholeslami A, Rezanejad M, Alipoor J (2023). Inertia augmentation-based optimal control strategy of a weak grid-connected microgrid with pv unit and energy storage system. J. Energy Stor..

[58] Kumar R, Bhende C (2023). A virtual adaptive rc damper control method to suppress voltage oscillation in dc microgrid. Int. J. Electr. Power Energy Syst..

[59] Tepe IF, Irmak E (2023). An integrated energy control system to provide optimum demand side management of a grid-interactive microgrid. Electr. Power Compon. Syst..

[60] Li Z (2023). Low-carbon operation method of microgrid considering carbon emission quota trading. Energy Rep..

[61] Ahmed I, Basit A, Rehan M, Hong K-S (2022). Multi-objective whale optimization approach for cost and emissions scheduling of thermal plants in energy hubs. Energy Rep..

[62] Taghizadegan N, Babaei F, Safari A (2023). A linear active disturbance rejection control technique for frequency control of networked microgrids. Energy Syst..

[63] Zhang G, Ge Y, Pan X, Zheng Y, Yang Y (2023). Hybrid robust-stochastic multi-objective optimization of combined cooling, heating, hydrogen and power-based microgrids. Energy.

[64] Martínez-Barbeito M, Gomila D, Colet P (2023). Dynamical model for power grid frequency fluctuations: Application to islands with high penetration of wind generation. IEEE Trans. Sustain. Energy.

[65] Dey B, Dutta S, Garcia Marquez FP (2023). Intelligent demand side management for exhaustive techno-economic analysis of microgrid system. Sustainability.

[66] Singh B, Bishnoi S, Sharma M, Singh P, Dhundhara S (2023). An application of nature inspried algorithm based dual-stage frequency control strategy for multi micro-grid system. Ain Shams Eng. J..

[67] de Araujo Silva Júnior W (2023). Characterization of the operation of a bess with a photovoltaic system as a regular source for the auxiliary systems of a high-voltage substation in brazil. Energies.

[68] Babak, B., Julia, M., Zia, E. & Chao, L. Guest editorial: Grid-forming converters placement and utilisation to enhance transmission and distribution performances under high penetration of inverter-based resources (2023).

[69] Khan KA, Atif A, Khalid M (2023). Hybrid battery-supercapacitor energy storage for enhanced voltage stability in dc microgrids using autonomous control strategy. Emerging Trends in Energy Storage Systems and Industrial Applications.

[70] Sikander A, Dheeraj A, Chatterjee A, Ahamad N (2022). Control design approach for improved voltage stability in microgrid energy storage system. Microsyst. Technol..

[71] Basati, A., Wu, J., Guerrero, J. M. & Vasquez, J. C. Internal model-based voltage control for dc microgrids under unknown external disturbances. In *2022 International Conference on Smart Energy Systems and Technologies (SEST)*, 1–6 (IEEE, 2022).

[72] Romero-L, M. *et al.* Analysis of supraharmonic emission in a microgrid in islanded and interconnected operation. In *2022 20th International Conference on Harmonics & Quality of Power (ICHQP)*, 1–6. 10.1109/ICHQP53011.2022.9808839 (2022).

[73] Alfalahi ST (2021). Supraharmonics in power grid: Identification, standards, and measurement techniques. IEEE Access.

[74] Naderi Y (2020). Power Quality Issues of Smart Microgrids: Applied Techniques and Decision Making Analysis. In Decision making applications in modern power systems.

[75] Tarasiuk T (2021). Review of power quality issues in maritime microgrids. IEEE Access.

[76] Wan Y, Dragičević T (2022). Data-driven cyber-attack detection of intelligent attacks in islanded dc microgrids. IEEE Trans. Ind. Electron..

[77] He Q, Shah P, Zhao X (2023). Resilient operation of dc microgrid against fdi attack: A gru based framework. Int. J. Electr. Power Energy Syst..

[78] Chen X, Zhou J, Shi M, Chen Y, Wen J (2022). Distributed resilient control against denial of service attacks in dc microgrids with constant power load. Renew. Sustain. Energy Rev..

[79] Ghafoori MS, Soltani J (2022). Designing a robust cyber-attack detection and identification algorithm for dc microgrids based on kalman filter with unknown input observer. IET Gener. Transm. Distrib..

[80] Baidya S, Nandi C (2022). A comprehensive review on dc microgrid protection schemes. Electr. Power Syst. Res..

[81] Basit A, Tufail M, Rehan M, Ahn CK (2023). Dynamic event-triggered approach for distributed state and parameter estimation over networks subjected to deception attacks. IEEE Trans. Signal Inf. Process. Netw..

[82] Ahmed I, Rehan M, Iqbal N, Ahn CK (2023). A novel event-triggered consensus approach for generic linear multi-agents under heterogeneous sector-restricted input nonlinearities. IEEE Trans. Netw. Sci. Eng..

[83] Zuo S, Pullaguramr D, Rajabinezhad M, Lewis FL, Davoudi A (2022). Resilient ac microgrids against correlated attacks. IEEE Access.

[84] Yang H, Deng C, Xie X, Ding L (2023). Distributed resilient secondary control for AC microgrid under FDI attacks. IEEE Trans. Circ. Syst. II Express Briefs.

[85] Ahmed I, Rehan M, Iqbal N (2022). A novel exponential approach for dynamic event-triggered leaderless consensus of nonlinear multi-agent systems over directed graphs. IEEE Trans. Circuits Syst. II Express Briefs.

[86] Sheng L (2022). Optimal communication network design of microgrids considering cyber-attacks and time-delays. IEEE Trans. Smart Grid.

[87] Mahmud, R. & Ingram, M. Background information on the protection requirements in ieee std 1547-2018. Tech. Rep., National Renewable Energy Lab.(NREL), Golden, CO (United States) (2022).

[88] Shi J, Ma L, Li C, Liu N, Zhang J (2022). A comprehensive review of standards for distributed energy resource grid-integration and microgrid. Renew. Sustain. Energy Rev..

[89] Basso TS, DeBlasio R (2004). Ieee 1547 series of standards: Interconnection issues. IEEE Trans. Power Electron..

[90] Ieee standard for interconnecting distributed resources with electric power systems. *IEEE Std 1547-2003* 1–28. 10.1109/IEEESTD.2003.94285 (2003).

[91] Rezvani MM, Mehraeen S, Ramamurthy JR, Field T (2020). Interaction of transmission-distribution system in the presence of der units-co-simulation approach. IEEE Open J. Ind. Appl..

[92] Photovoltaics DG, Storage E (2018). Ieee standard for interconnection and interoperability of distributed energy resources with associated electric power systems interfaces. IEEE Std..

[93] Rana MM, Li L, Su SW (2017). Cyber attack protection and control of microgrids. IEEE/CAA J. Autom. Sin..

[94] Priyadharshini N, Gomathy S, Sabarimuthu M (2020). A review on microgrid architecture, cyber security threats and standards. Mater. Today Proc..

[95] Espina E (2020). Distributed control strategies for microgrids: An overview. IEEE Access.

[96] Gu Y, Xiang X, Li W, He X (2013). Mode-adaptive decentralized control for renewable dc microgrid with enhanced reliability and flexibility. IEEE Trans. Power Electron..

[97] Yamashita DY, Vechiu I, Gaubert J-P (2020). A review of hierarchical control for building microgrids. Renew. Sustain. Energy Rev..

[98] Taft, J. D. Comparative architecture analysis: Using laminar structure to unify multiple grid architectures. Tech. Rep., Pacific Northwest National Lab.(PNNL), Richland, WA (United States) (2016).

[99] Vasquez JC, Guerrero JM, Miret J, Castilla M, De Vicuna LG (2010). Hierarchical control of intelligent microgrids. IEEE Ind. Electron. Mag..

[100] Pires VF, Cordeiro A, Foito D, Silva JF (2022). Control transition mode from voltage control to mppt for pv generators in isolated dc microgrids. Int. J. Electr. Power Energy Syst..

[101] Saifudheen, P. & Thresia, M. A droop controller based active power sharing of parallel inverter islanded microgrid. In *2022 International Conference on Futuristic Technologies in Control Systems & Renewable Energy (ICFCR)*, 1–6 (IEEE, 2022).

[102] Kolluri RR (2017). Power sharing in angle droop controlled microgrids. IEEE Trans. Power Syst..

[103] D’Arco S, Suul JA (2013). Equivalence of virtual synchronous machines and frequency-droops for converter-based microgrids. IEEE Trans. Smart Grid.

[104] Eskandari M, Li L, Moradi MH (2017). Decentralized optimal servo control system for implementing instantaneous reactive power sharing in microgrids. IEEE Trans. Sustain. Energy.

[105] Eisapour-Moarref A, Kalantar M, Esmaili M (2019). Power sharing in hybrid microgrids using a harmonic-based multidimensional droop. IEEE Trans. Ind. Inf..

[106] Wu X, Shen C, Iravani R (2016). Feasible range and optimal value of the virtual impedance for droop-based control of microgrids. IEEE Trans. Smart Grid.

[107] Nasirian, V., Davoudi, A. & Lewis, F. L. Distributed adaptive droop control for dc microgrids. In *2014 IEEE Applied Power Electronics Conference and Exposition-APEC 2014*, 1147–1152 (IEEE, 2014).

[108] Alam, F., Ashfaq, M., Zaidi, S. S. & Memon, A. Y. Robust droop control design for a hybrid ac/dc microgrid. In *2016 UKACC 11th International Conference on Control (CONTROL)*, 1–6 (IEEE, 2016).

[109] Zheng H (2023). An islanding detection method using synchronized small-ac-signal injection for grid-forming inverters in microgrids. IEEE Trans. Power Electron..

[110] Weise B (2015). Impact of k-factor and active current reduction during fault-ride-through of generating units connected via voltage-sourced converters on power system stability. IET Renew. Power Gener..

[111] Zuo Y (2021). Performance assessment of grid-forming and grid-following converter-interfaced battery energy storage systems on frequency regulation in low-inertia power grids. Sustain. Energy Grids Netw..

[112] Ahmethodzic L, Music M (2021). Comprehensive review of trends in microgrid control. Renew. Energy Focus.

[113] Rani VU, Divya A, Vinay A, Charan KS, Kumar IJ (2023). A review on decentralized control techniques in a microgrid using various hybrid energy storage systems. J. Pharm. Neg. Res..

[114] Behera MK, Saikia LC (2023). A novel spontaneous control for autonomous microgrid vsc system using bpf droop and improved hysteresis band control scheme. Electr. Power Syst. Res..

[115] Shi M (2019). Pi-consensus based distributed control of ac microgrids. IEEE Trans. Power Syst..

[116] Zhou J, Zhang H, Sun Q, Ma D, Huang B (2017). Event-based distributed active power sharing control for interconnected ac and dc microgrids. IEEE Trans. Smart Grid.

[117] He J, Li Y, Liang B, Wang C (2017). Inverse power factor droop control for decentralized power sharing in series-connected-microconverters-based islanding microgrids. IEEE Trans. Ind. Electron..

[118] Malik SM, Ai X, Sun Y, Zhengqi C, Shupeng Z (2017). Voltage and frequency control strategies of hybrid ac/dc microgrid: A review. IET Gener. Transm. Distrib..

[119] Daniel A, Dayalan S (2022). Effective communication-based reactive power sharing scheme for meshed microgrid in an islanded mode. J. Circ. Syst. Comput..

[120] Bidgoli MA, Ahmadian A (2022). Multi-stage optimal scheduling of multi-microgrids using deep-learning artificial neural network and cooperative game approach. Energy.

[121] Mohammadi E, Alizadeh M, Asgarimoghaddam M, Wang X, Simões MG (2022). A review on application of artificial intelligence techniques in microgrids. IEEE J. Emerg. Sel. Top. Ind. Electron..

[122] Zheng Z, Yang S, Guo Y, Jin X, Wang R (2023). Meta-heuristic techniques in microgrid management: A survey. Swarm Evol. Comput..

[123] Zhang B (2023). Source-storage-load coordinated master-slave control strategy for islanded microgrid considering load disturbance and communication interruption. IEEE Trans. Cybern..

[124] Alabdullah MH, Abido MA (2022). Microgrid energy management using deep q-network reinforcement learning. Alex. Eng. J..

[125] Zhu J (2022). Optimal scheduling of a wind energy dominated distribution network via a deep reinforcement learning approach. Renew. Energy.

[126] Nguyen TL, Nguyen HT, Wang Y, Mohammed OA, Anagnostou E (2022). Distributed secondary control in microgrids using synchronous condenser for voltage and frequency support. Energies.

[127] Wang Z (2022). Distributed event-triggered fixed-time fault-tolerant secondary control of islanded ac microgrid. IEEE Trans. Power Syst..

[128] Lu X, Lai J (2021). Communication constraints for distributed secondary control of heterogeneous microgrids: A survey. IEEE Trans. Ind. Appl..

[129] Zhu, Y., Zhuo, F. & Xiong, L. Communication platform for energy management system in a master-slave control structure microgrid. In *Proceedings of The 7th International Power Electronics and Motion Control Conference*, vol. 1, 141–145 (IEEE, 2012).

[130] Liu W, Xu Y, Feng X, Wang Y (2022). Optimal fuzzy logic control of energy storage systems for v/f support in distribution networks considering battery degradation. Int. J. Electr. Power Energy Syst..

[131] Zhao C, Li X (2023). Microgrid optimal energy scheduling considering neural network based battery degradation. IEEE Trans. Power Syst..

[132] Qian T, Liu Y, Zhang W, Tang W, Shahidehpour M (2019). Event-triggered updating method in centralized and distributed secondary controls for islanded microgrid restoration. IEEE Trans. Smart Grid.

[133] Lee J-W, Kim M-K, Kim H-J (2021). A multi-agent based optimization model for microgrid operation with hybrid method using game theory strategy. Energies.

[134] Tazi K, Abbou FM, Abdi F (2020). Multi-agent system for microgrids: Design, optimization and performance. Artif. Intell. Rev..

[135] Poonahela, I. *et al.* Predictive voltage and frequency restoration for decentralized fcs-mpc based droop controlled dgs in ac microgrids. In *2022 3rd International Conference on Smart Grid and Renewable Energy (SGRE)*, 1–6 (IEEE, 2022).

[136] Mirzabeigi A, Kazemy A, Ramezani M, Azimi SM (2023). Design of a secondary controller based on distributed cooperative control of distributed generators (dgs) with multi-agent systems approach considering dos cyber attacks. Nashriyyah-i Muhandisi-i Barq va Muhandisi-i Kampyutar-i Iran.

[137] Qin, Q., Liu, S. & Fu, W. Tertiary control based on non-parametric model prediction for dc microgrid cluster. In *2021 IEEE International Conference on Predictive Control of Electrical Drives and Power Electronics (PRECEDE)*, 798–803 (IEEE, 2021).

[138] Zhang C, Dou X, Wang L, Dong Y, Ji Y (2022). Distributed cooperative voltage control for grid-following and grid-forming distributed generators in islanded microgrids. IEEE Trans. Power Syst..

[139] Zhao E (2022). Accurate peer-to-peer hierarchical control method for hybrid dc microgrid clusters. Energies.

[140] Panda SK, Ghosh A (2020). A computational analysis of interfacing converters with advanced control methodologies for microgrid application. Technol. Econ. Smart Grids Sustain. Energy.

[141] Andishgar MH, Gholipour M, Hooshmand R-A (2020). Improved secondary control for optimal unbalance compensation in islanded microgrids with parallel dgs. Int. J. Electr. Power Energy Syst..

[142] Alahmed AS, Al-Muhaini MM (2020). An intelligent load priority list-based integrated energy management system in microgrids. Electr. Power Syst. Res..

[143] Zhang Z, Zuo Z, Wang Y (2022). Distributed control for state-of-charge balance and load voltage regulation in dc microgrids with clustered generations. Asian J. Control.

[144] Singh C, Shimi S, Mathew L (2022). Power quality enhancement of dc microgrid: A review. Acta Energet..

[145] Sharma J, Sundarabalan C, Sitharthan R, Balasundar C, Srinath N (2022). Power quality enhancement in microgrid using adaptive affine projection controlled medium voltage distribution static compensator. Sustain. Energy Technol. Assess..

[146] Yaramasu V, Wu B (2016). Model Predictive Control of Wind Energy Conversion Systems.

[147] Basit A, Tufail M, Rehan M, Ahmed I (2023). A new event-triggered distributed state estimation approach for one-sided Lipschitz nonlinear discrete-time systems and its application to wireless sensor networks. ISA Trans..

[148] Basit, A., Tufail, M., Hong, K.-S., Rehan, M. & Ahmed, I. Event-triggered distributed exponential $${H}_\infty $$ observers design for discrete-time nonlinear systems over wireless sensor networks. In *2022 13th Asian Control Conference (ASCC)*, 1730–1735 (2022).

[149] Salehirad M, MollaieEmamzadeh M (2023). Energy management and harmonic compensation of micro-grids via multi-agent systems based on decentralized control architecture. IET Renew. Power Gener..

[150] Wang J, Deng X, Guo J, Zeng Z (2023). Resilient consensus control for multi-agent systems: A comparative survey. Sensors.

[151] Pamulapati T (2022). A review of microgrid energy management strategies from the energy trilemma perspective. Energies.

[152] Zhang Z (2022). Advances and opportunities in the model predictive control of microgrids: Part i-primary layer. Int. J. Electr. Power Energy Syst..

[153] Zhao R, Miao M, Ju Y (2022). Trends of optimal dispatching of microgrid for fishery based on model predictive control. Inf. Process. Agric..

[154] Badar AQ, Anvari-Moghaddam A (2022). Smart home energy management system-a review. Adv. Build. Energy Res..

[155] Aloo, L. A., Kihato, P. K., Kamau, S. I. & Orenge, R. S. Model predictive control-adaptive neuro-fuzzy inference system control strategies for photovoltaic-wind microgrid: Feasibility review. *2020 IEEE PES/IAS PowerAfrica* 1–5 (2020).

[156] Babayomi O, Zhang Z, Dragicevic T, Hu J, Rodriguez J (2023). Smart grid evolution: Predictive control of distributed energy resources-a review. Int. J. Electr. Power Energy Syst..

[157] Adetokun BB, Oghorada O, Abubakar SJ (2022). Superconducting magnetic energy storage systems: Prospects and challenges for renewable energy applications. J. Energy Stor..

[158] Das P, Chanda S, De A (2020). Artificial intelligence-based economic control of micro-grids: A review of application of iot. Comput. Adv. Commun. Circ. Syst. Proc. ICCACCS.

[159] Rodríguez F, Fleetwood A, Galarza A, Fontán L (2018). Predicting solar energy generation through artificial neural networks using weather forecasts for microgrid control. Renew. Energy.

[160] Velik R, Nicolay P (2014). A cognitive decision agent architecture for optimal energy management of microgrids. Energy Convers. Manage..

[161] Jafari, M. *et al.* Adaptive neural network based intelligent secondary control for microgrids. In *2018 IEEE Texas Power and Energy Conference (TPEC)*, 1–6 (IEEE, 2018).

[162] Yin L, Yu T, Yang B, Zhang X (2019). Adaptive deep dynamic programming for integrated frequency control of multi-area multi-microgrid systems. Neurocomputing.

[163] Goh HH (2022). An assessment of multistage reward function design for deep reinforcement learning-based microgrid energy management. IEEE Trans. Smart Grid.

[164] Chen D (2021). Powernet: Multi-agent deep reinforcement learning for scalable powergrid control. IEEE Trans. Power Syst..

[165] Chen P, Liu S, Chen B, Yu L (2022). Multi-agent reinforcement learning for decentralized resilient secondary control of energy storage systems against dos attacks. IEEE Trans. Smart Grid.

[166] Warraich Z, Morsi W (2023). Early detection of cyber-physical attacks on fast charging stations using machine learning considering vehicle-to-grid operation in microgrids. Sustain. Energy Grids Netw..

[167] De Persis C, Weitenberg ER, Dörfler F (2018). A power consensus algorithm for dc microgrids. Automatica.

[168] Basit A, Tufail M, Rehan M (2022). An adaptive gain based approach for event-triggered state estimation with unknown parameters and sensor nonlinearities over wireless sensor networks. ISA Trans..

[169] Ahmed, I., Rehan, M., Hong, K.-S. & Basit, A. Event-triggered leaderless robust consensus control of nonlinear multi-agents under disturbances. In *2022 13th Asian Control Conference (ASCC)*, 1736–1741 (2022).

[170] Alvi U-E-H (2022). A novel incremental cost consensus approach for distributed economic dispatch over directed communication topologies in a smart grid. Soft. Comput..

